# Self-inflicted intracranial self-injury

**DOI:** 10.4103/0974-2700.76814

**Published:** 2011

**Authors:** Matthew M Large, Olav B Nielssen, Nicholas Babidge

**Affiliations:** School of Psychiatry, University of New South Wales, Sydney, New South Wales, Australia; 1 Clinical Research Unit for Anxiety and Depression, St Vincent’s Hospital, Sydney, Australia; 2 Mental Health Service, Sutherland Hospital, New South Wales, Australia

Sir,

We were interested to read the recent case history of a 22-year-old man with schizophrenia who hammered a large nail through his cranium into his frontal lobe.[1] The authors correctly state that this form of injury is rare. However, intracranial self-stabbing (ICSS) might not be as rare as one might believe. In a survey of severe mutilation in New South Wales, Australia, we found five similar cases among 7 million population of over a ten-year period.[2,3] Thus, ICSS seems to be no more rare than the well-known phenomenon of self-enucleation.[4] Furthermore, a search of the medical literature revealed 47 earlier accounts of manually self-inflicted intracranial injuries, excluding those involving firearms and other trigger operated devices such as spear and nail guns.[3] Although the most common site of entry into the brain in our series was the orbital space, some hammered through bone like the patient in the recent case and many of the 52 ICSS cases were similar to the recent case with respect to male sex, young age and the presence of a psychotic illness such as schizophrenia. The recent case is also similar to most of those in our series because the outcome for the patient was not dire, presumably due to the low energy involved (when compared to high-velocity gunshot injury) and the type of thoughtful neurosurgical treatment described by the authors. The authors also note that these bizarre self-destructive acts are not always motivated by a wish to die, and might sometimes be motivated by the delusional belief that the self-injury will be helpful to the patient, perhaps by releasing evil or stopping voices. However, we found that in all but a few ICSS cases the patients were unable to provide an account of their motivations. The term self-mutilation is usually used to describe serious damage to the person’s body as a direct result of the self’s deliberate nonsuicidal actions.[5] In addition to being a medical emergency and a bizarre and interesting behavior, we believe ICSS can be regarded as the most pure form of self-mutilation because the self is both the perpetrator and the victim.
